# Genome Sequence of a Bovine Isolate of Pasteurella multocida Strain 232

**DOI:** 10.1128/MRA.00333-19

**Published:** 2019-05-16

**Authors:** Sahlu Ayalew, Anthony W. Confer, Matthew Brian Couger

**Affiliations:** a Department of Veterinary Pathobiology, CVHS, Oklahoma State University, Stillwater, Oklahoma, USA; b High Performance Computing Center, Oklahoma State University, Stillwater, Oklahoma, USA; University of Arizona

## Abstract

We present here the genome sequence of Pasteurella multocida 232, a bacterium that is associated with pneumonia in humans as well as in many animal species. The genome of Pasteurella multocida 232 has an *N*_50_ value of 187.32 kb and a total size of 2.34 Mb.

## ANNOUNCEMENT

Pasteurella multocida 232 is a Gram-negative bacterium that can cause disease in many animal species, including humans (1). In cattle, P. multocida 232 is one of the more common bacteria associated with pneumonia and is one of several important pathogenic bacteria involved in the bovine respiratory disease complex, the major cause of disease and economic losses in the beef cattle industry (2–4). In our laboratory, we conducted numerous studies on P. multocida 232, including on characterization of outer membrane proteins, immunogenicity, and pathogenicity in mice and cattle (5–10). In most of those studies, P. multocida strain 232 was used. The strain was originally isolated from a case of calf pneumonia and was kindly provided to us by John Berg (now deceased) at the University of Missouri. The isolate was typed as serogroup A and serotype 3 and was an ideal strain for studying aspects of bovine respiratory disease pathogenesis and immunity because it is highly immunogenic and pathogenic in both cattle and mice.

Individual colonies from overnight culture of P. multocida 232 on brain heart infusion (BHI) agar supplemented with 5% defibrinated sheep blood (Hardy Diagnostics, Santa Maria, CA, USA) were transferred into culture tubes, each containing 5 ml of BHI broth (Becton, Dickinson and Company, Sparks, MD, USA) and incubated in a shaker incubator at 37°C. The overnight starter culture was transferred into Erlenmeyer flasks with 50 ml BHI and incubated until the mid-log phase in a shaker incubator. Bacterial cells were harvested by centrifugation at 12,000 × *g* for 20 min at 4°C. Genomic DNA was extracted from the pellet using a Wizard genomic DNA purification kit (Promega, Madison, WI, USA) with some modifications. Capsular material was removed by repeated extraction with buffered phenol, phenol-chloroform-isoamyl alcohol, and chloroform-isoamyl alcohol, and genomic DNA was precipitated with 95% ethanol in the presence of sodium acetate. Each pellet was washed with ice-cold 70% ethanol. The quality of genomic DNA was confirmed by agarose gel electrophoresis and a 260/280 ratio of 1.8 to 1.9.

Genomic DNA from P. multocida 232 was sequenced on the Illumina HiSeq platform using 2 × 150-bp paired-end reads. A total of 4,626,697 paired-end reads (1.39 Gb) were produced. After quality filtering, the sequence data were generated using the standard Illumina protocol recommended by the sequence provider (Novogene). Quality-filtered reads were subsampled to approximately 200× coverage for assembly. These reads were assembled with the short-read De Bruijn graph assembly program Velvet (11). The Velvet assembly runtime settings used in the assembly were a kmer value of 105, filtering to remove all the contigs that were not supported by at least 7× coverage, and an expected coverage value of 200×. The resulting assembly had an *N*_50_ scaffold size of 187,320 bp, a maximum scaffold size of 525,608 bp, and a total of 2,232,239 bp. Initial gene models for analysis and quality of assembly evaluation were produced using the Prodigal version 2.6.3 (12) prokaryotic gene-calling program. Runtime Prodigal settings were the standard software settings recommended for complete genomes. Prodigal produced 2,054 protein-coding gene models in total. All predicted protein sequences derived from the assembly were functionally annotated using a combination of homology and conserved domain search using NCBI BLAST+ version 2.8.1 (13) and HMMER version 3.0 (14) against the formatted Pfam 32.0 database. Standard recommended settings were used for both BLAST and hmmscan. Public gene models associated with the accession were generated with the NCBI Prokaryotic Genome Annotation Pipeline (15).

## 

### Data availability.

This whole-genome shotgun project has been deposited at DDBJ/ENA/GenBank under the accession number SJDX00000000. The version described in this paper is the first version, SJDX01000000. The sequences have been submitted to the Sequence Read Archive (SRA) under the accession number SRX5524447.
